# Oncobiotics in urinary bladder cancer. A narrative review of living cancer therapeutics

**DOI:** 10.3389/fonc.2026.1845015

**Published:** 2026-06-22

**Authors:** Suleiman Abuhasanein

**Affiliations:** 1 Department of Urology, Institute of Clinical Science, Sahlgrenska Academy, University of Gothenburg, Gothenburg, Sweden; 2 Department of Surgery, Urology section, NÄL and Uddevalla (NU)-Hospital Group, Uddevalla, Region Västra Götaland, Sweden; 3 Department of Research and Development, NU-Hospital Group, Trollhättan, Region Västra Götaland, Sweden

**Keywords:** bacteria, bladder cancer, immunotherapy, living cancer therapeutics, oncobiotics, oncolytic viruses, probiotics

## Abstract

Urinary bladder cancer (UBC) remains a major global health burden, with high recurrence rates and limited therapeutic options for patients who fail standard intravesical and systemic treatments. In recent years, Living Cancer Therapeutics (LCTs)—including bacteria-, virus-, and microbiome-based oncobiotics—emerged as innovative biological strategies capable of overcoming key limitations of conventional cancer therapies. This article is a narrative review aimed at mapping the mechanistic landscape, historical development, and translational progress of LCTs in UBC. Five interrelated mechanisms were identified through which oncobiotics exert therapeutic effects: (i) direct tumor destruction via bacterial colonization, cytolysis, and metabolic deprivation; (ii) immune system modulation through innate and adaptive immune activation; (iii) engineered drug delivery and synthetic biology enabling programmable, tumor-restricted payload release; (iv) oncolytic virotherapy combining selective tumor lysis with immune priming; and (v) microbiome-driven immune modulation influencing treatment responsiveness. Although conceptually distinct, these mechanisms frequently overlap in practice, reflecting the multifunctional nature of living therapeutics. Clinical translation has progressed furthest for immune-mediated approaches such as Bacillus Calmette–Guérin (BCG) and selected oncolytic viral platforms, particularly in BCG-unresponsive UBC, although current evidence remains limited by small studies, heterogeneous endpoints, and insufficient long-term follow-up. Advances in genetic engineering and synthetic biology have enabled the development of increasingly sophisticated investigational platforms, including engineered oncolytic viruses, programmable bacterial vectors, and microbiome-based therapeutic strategies; however, most remain at an early preclinical or translational stage. UBC may represent a favorable setting for LCT development due to the accessibility of the bladder and the established use of intravesical therapies, although delivery efficiency and therapeutic durability remain important challenges. Despite encouraging early findings, significant limitations persist, including biological delivery barriers, host immune neutralization, interpatient heterogeneity, biosafety concerns, regulatory complexity, and the scarcity of late-phase randomized clinical data. Further translational research, biomarker development, and long-term clinical evaluation will therefore be required to determine the future role of LCTs in UBC management.

## 1 Introduction

Urinary bladder cancer (UBC) was among the most prevalent cancers worldwide, ranking seventh by five-year prevalence in 2020, with more than 1.7 million people living within five years of diagnosis (1). UBC is recognized as the cancer entailing the highest lifetime treatment costs per patient (2, 3). The risk of UBC increases with age, with most cases diagnosed in individuals over 55 years (4), and tobacco smoking represents the predominant risk factor, accounting for approximately half of all cases and 37% of disease-related mortality (5).

Like other malignancies, UBC develops through multiple oncogenic mechanisms, including sustained proliferative signaling, evasion of growth suppressors, resistance to cell death, replicative immortality, angiogenesis, metabolic reprogramming, and immune evasion (6). Despite multimodal management incorporating surgery, radiotherapy, intravesical therapy, systemic chemotherapy, and immunotherapy, treatment options for advanced or recurrent UBC following failure of standard therapies remain limited by significant drug resistance, poor efficacy, and severe off-target toxicity (7). These limitations underscore the urgent need for innovative therapeutic strategies.

Cancer therapy is entering an emerging therapeutic field with the emergence of “living cancer therapeutics” (LCTs). In this review, LCTs are defined as living or biologically active microorganisms and microorganism-derived platforms—including bacteria, oncolytic viruses, engineered microbial systems, and microbiome-modulating agents—that exert anticancer effects through selective tumor targeting, modulation of antitumor immunity, delivery of therapeutic payloads, or alteration of the tumor microenvironment (8–10). The most established clinical example is Bacillus Calmette-Guérin (BCG), which is the gold standard intravesical adjuvant immunotherapy for high-grade non-muscle-invasive bladder cancer (NMIBC) (11). Beyond BCG, bacteria offer several attractive therapeutic properties, including selective tumor colonization, and immune modulation (12). However, effective anticancer strains must be safe, genetically engineerable, and capable of tumor invasion, and direct tumor cell lysis while sparing healthy tissue (13). In parallel, oncolytic viruses—either naturally occurring or genetically engineered, represent a promising class of LCTs that selectively replicate within malignant cells, inducing tumor lysis and stimulating antitumor immune responses (14). Although biologically diverse, these platforms share several defining characteristics: they employ living or biologically active microbial systems capable of tumor-selective activity, immune modulation, or programmable therapeutic delivery. Accordingly, the term LCTs is used herein as a functional and conceptual framework rather than a strict mechanistic or taxonomic classification.

From historical observations of infection-associated tumor regression to contemporary bioengineering advances, this narrative review was conducted using a targeted, exploratory search of the PubMed database to identify representative preclinical and clinical studies on LCTs in UBC. A narrative review approach was chosen because the field of LCTs in UBC remains highly heterogeneous and rapidly evolving, encompassing a wide range of preclinical, translational, and early-phase clinical studies. Accordingly, the primary aim of this review was to provide a broad conceptual and translational overview of the field, with emphasis on mechanistic diversity, emerging therapeutic strategies, and current clinical development, rather than to perform a systematic evidence synthesis or quantitative meta-analysis.

Therefore, the review was not intended to be systematic or exhaustive, but rather to synthesize key mechanistic concepts, historical milestones, and emerging translational advances across bacterial, viral, and microbiome-based platforms. Both preclinical and clinical investigations were included to provide a comprehensive conceptual overview of the field and to highlight emerging therapeutic strategies relevant to UBC. Studies were selected based on relevance to bladder cancer, mechanistic insight, and translational or clinical applicability. However, no formal risk-of-bias assessment of included studies was performed.

**Figure 1:**
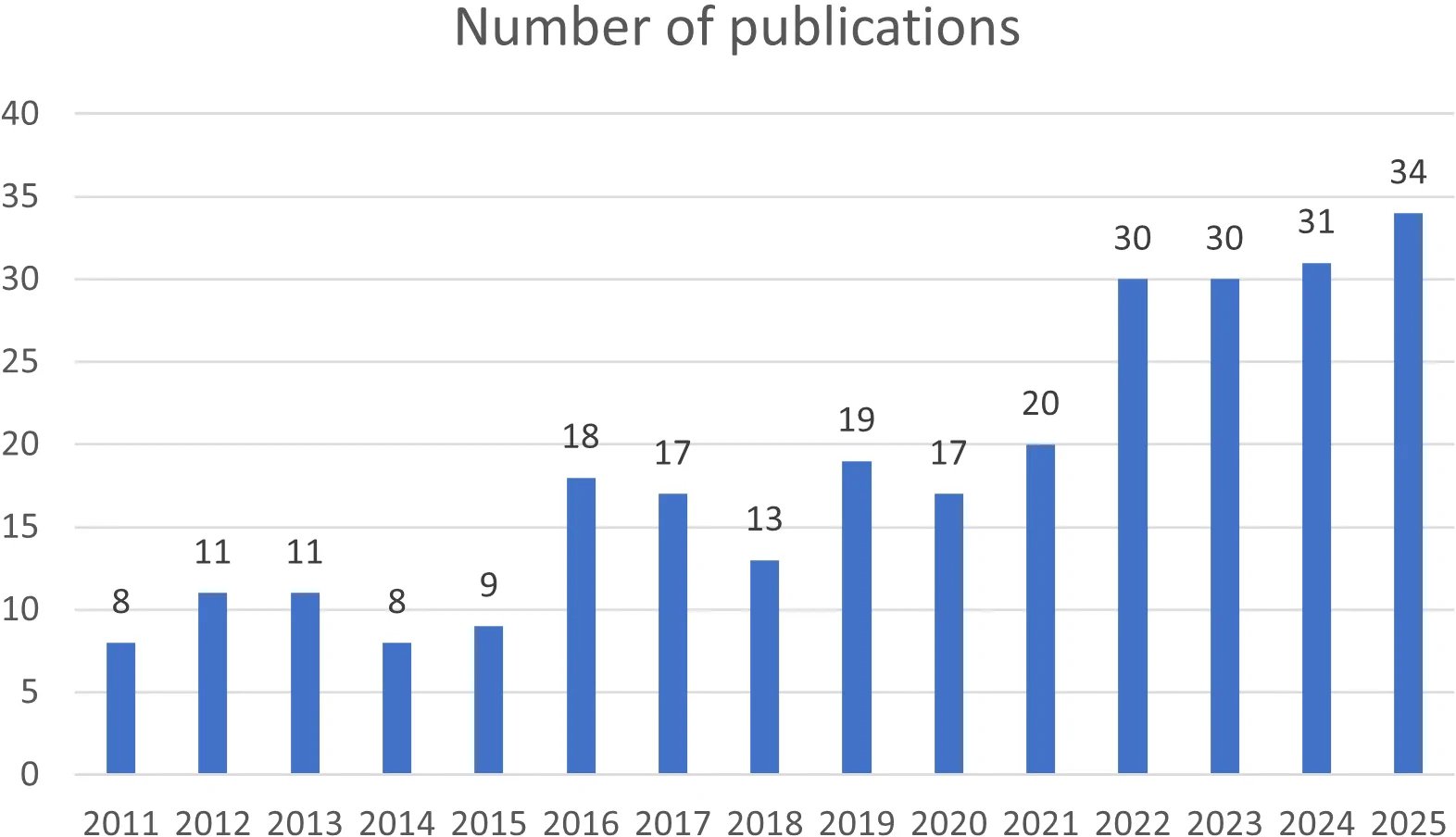
Number of publications using living cancer therapeutic with different types of oncobiotics of UBC from the last 15 years (2011-2025). Publication numbers were calculated using the PubMed database (2026, Jan 1^st^) using the keywords (oncobiotic OR "living cancer therapeutic" OR "live cancer therapeutic" OR "living therapeutic" OR "live biotherapeutic product" OR "oncolytic virus" OR virotherapy OR bacteriotherapy OR "bacterial therapy" OR "immune-modulating bacteria" ) AND ( "urinary bladder cancer"[MeSH] OR "bladder cancer" OR "bladder carcinoma" OR "urothelial carcinoma" ).

Search terms included combinations of “bladder cancer,” “urinary bladder cancer,” “living cancer therapeutics,” “oncobiotics,” “bacteria,” “oncolytic virus,” “BCG,” “gene therapy,” “microbiome,” and related keywords. Trends in scientific interest over the past decade are illustrated in **Figure 1**.

## 2 Historical view

The conceptual foundations of LCTs can be traced back to the 19th century, when Louis Pasteur and Robert Koch established germ theory and transformed the understanding of microorganisms in human disease. Since then, accumulating evidence has demonstrated that microbial composition and activity influence a broad spectrum of pathological conditions, including metabolic disorders, inflammatory diseases, and cancer (15, 16). Early clinical observations suggested that infections could occasionally induce tumor regression, hinting at the therapeutic potential of microbes (17).

In the late 19th century, Wilhelm Busch and Friedrich Fehleisen independently reported tumor regression in cancer patients who developed erysipelas caused by *Streptococcus pyogenes* (18). These findings inspired deliberate infection-based interventions, most notably by William Coley, who demonstrated that intratumoral administration of heat-inactivated *S. pyogenes* and *Serratia marcescens*—later known as Coley’s toxins—could suppress tumor growth and, in some cases, lead to durable clinical responses (19). Parallel efforts by investigators such as T. Doyen and Livingston-Wheeler further explored microbial and vaccine-based anticancer approaches, laying early groundwork for biologically driven cancer therapy (20, 21).

The immunotherapeutic potential of microbes was firmly established in 1976 when Morales and colleagues demonstrated the efficacy of intravesical BCG in superficial UBC. BCG remains the most successful example of microbial immunotherapy and continues to serve as the standard of care for high-grade NMIBC (11).

**Figure 2:**
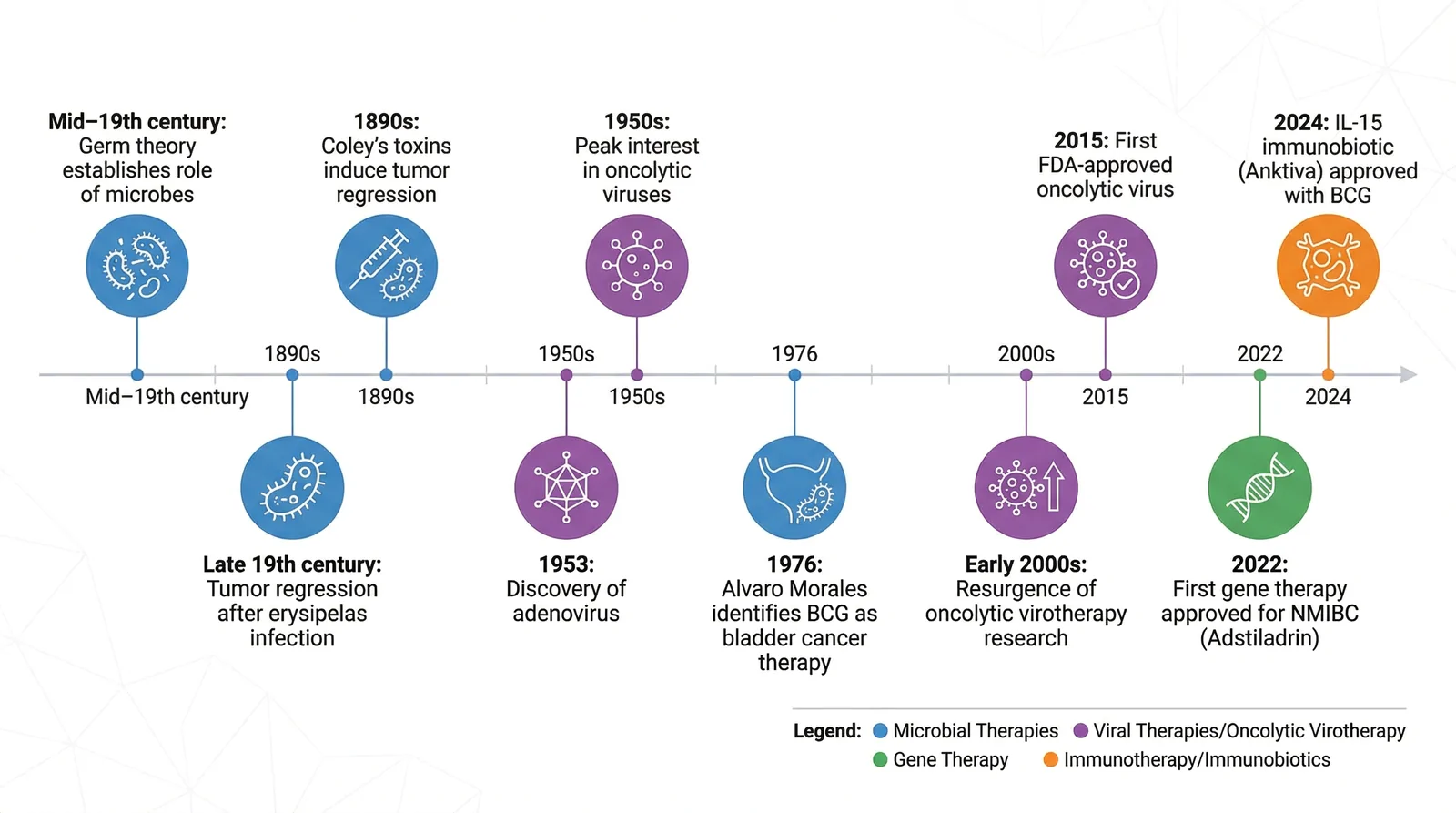
The evolution and the timeline of major discoveries for LCTs.

Interest in viral-based cancer therapies followed a similar trajectory, with early enthusiasm in the mid-20th century, subsequent decline, and renewed resurgence in the early 2000s as genetic engineering enabled tumor-selective viral replication (22). Regulatory milestones subsequently validated this approach. The FDA approval of the first oncolytic virus talimogene laherparepvec in 2015 (for melanoma) (23), nadofaragene firadenovec (Adstiladrin) in 2022, -as the first approved gene therapy for BCG-unresponsive NMIBC- (24), and nogapendekin alfa inbakicept (Anktiva) in 2024 -an IL-15 receptor agonist used in combination with BCG for patients with BCG-unresponsive disease (25) collectively underscore the clinical maturation of LCTs. The historical development of living cancer therapeutics and key scientific and regulatory milestones are summarized in **Figure 2**. An overview of LCTs for UBC discussed in this review are summarized in **Table 1**.

**Table 1:** Overview of living cancer therapeutics in urinary bladder cancer.

Therapeutic platform	Type of LCT	Mechanism of action	Route of administration	Immune effects	Developmental stage	Translational/clinical status
BCG (Bacillus Calmette–Guérin)	Live attenuated bacteria	Activation of innate and adaptive antitumor immunity	Intravesical	Strong activation of macrophages, NK cells, dendritic cells, and T cells	Clinically established	Current standard of care for high-risk NMIBC
CG0070	Oncolytic adenovirus expressing GM-CSF	Selective replication in Rb-deficient tumor cells with GM-CSF-mediated immune activation	Intravesical	Promotes immunogenic cell death and local immune stimulation	Phase II/III	Promising efficacy in BCG-unresponsive NMIBC
CVA21 (CAVATAK)	Oncolytic coxsackievirus	Selective viral replication and tumor lysis via ICAM-1 targeting	Intravesical	Induces tumor inflammation and immune gene upregulation	Phase I	Early clinical proof-of-concept established
Nadofaragene firadenovec	Adenoviral gene therapy	Delivery of IFN-α2b gene to urothelial cells	Intravesical	Enhances interferon-mediated antitumor immunity	FDA-approved	Approved for BCG-unresponsive NMIBC
OH2	Genetically modified HSV-2	Direct oncolysis combined with GM-CSF-mediated immune activation	Intratumoral / intravesical	Enhances immune cell recruitment and activation	Phase I/II	Under clinical evaluation
MV-NIS	Oncolytic measles virus	Selective viral infection and tumor destruction	Intravesical / intravenous	Promotes immune infiltration and tumor inflammation	Early clinical development	Under clinical evaluation
Lactobacillus spp. (LGG, L. casei)	Probiotic bacteria	Immune modulation and enhancement of antitumor immune signaling	Oral and intravesical	Enhances macrophage and neutrophil recruitment	Preclinical and clinical adjunct studies	Potential adjunctive immunomodulatory strategy
Engineered Salmonella spp.	Genetically modified bacterial vector	Tumor-selective delivery of siRNA and immunomodulatory payloads	Intravesical / experimental systemic administration	Increases CD8+ T-cell infiltration and immune activation	Preclinical	Promising programmable delivery platform
Bifidobacterium infantis–HSV-TK system	Engineered probiotic suicide-gene therapy	HSV-TK/ganciclovir-mediated induction of tumor apoptosis	Experimental intravesical delivery	Indirect immune activation secondary to tumor apoptosis	Preclinical	Proof-of-concept demonstrated in animal models
IL-15–engineered Salmonella VNP20009	Synthetic bacterial immunotherapy	Localized IL-15/IL-15Rα cytokine delivery	Intravesical	Strong activation of NK cells and CD8+ T cells	Preclinical	High translational potential for immune enhancement
MCNA (Mycobacterium phlei cell wall–nucleic acid complex)	Bacterial-derived immunotherapy	Immune stimulation and pro-apoptotic activity	Intravesical	Enhances local immune activation	Early clinical evaluation	Investigated in BCG-unresponsive NMIBC
Bacteriobots (engineered E. coli Nissle 1917)	Bioengineered bacterial microrobots	Targeted tumor localization and ROS-mediated tumor destruction	Experimental local delivery	Potential secondary immune activation	Early preclinical	Emerging next-generation precision platform

## 3 LCT-mediated mechanisms of oncobiotics in UBC-oncotherapy

**Figure 3:**
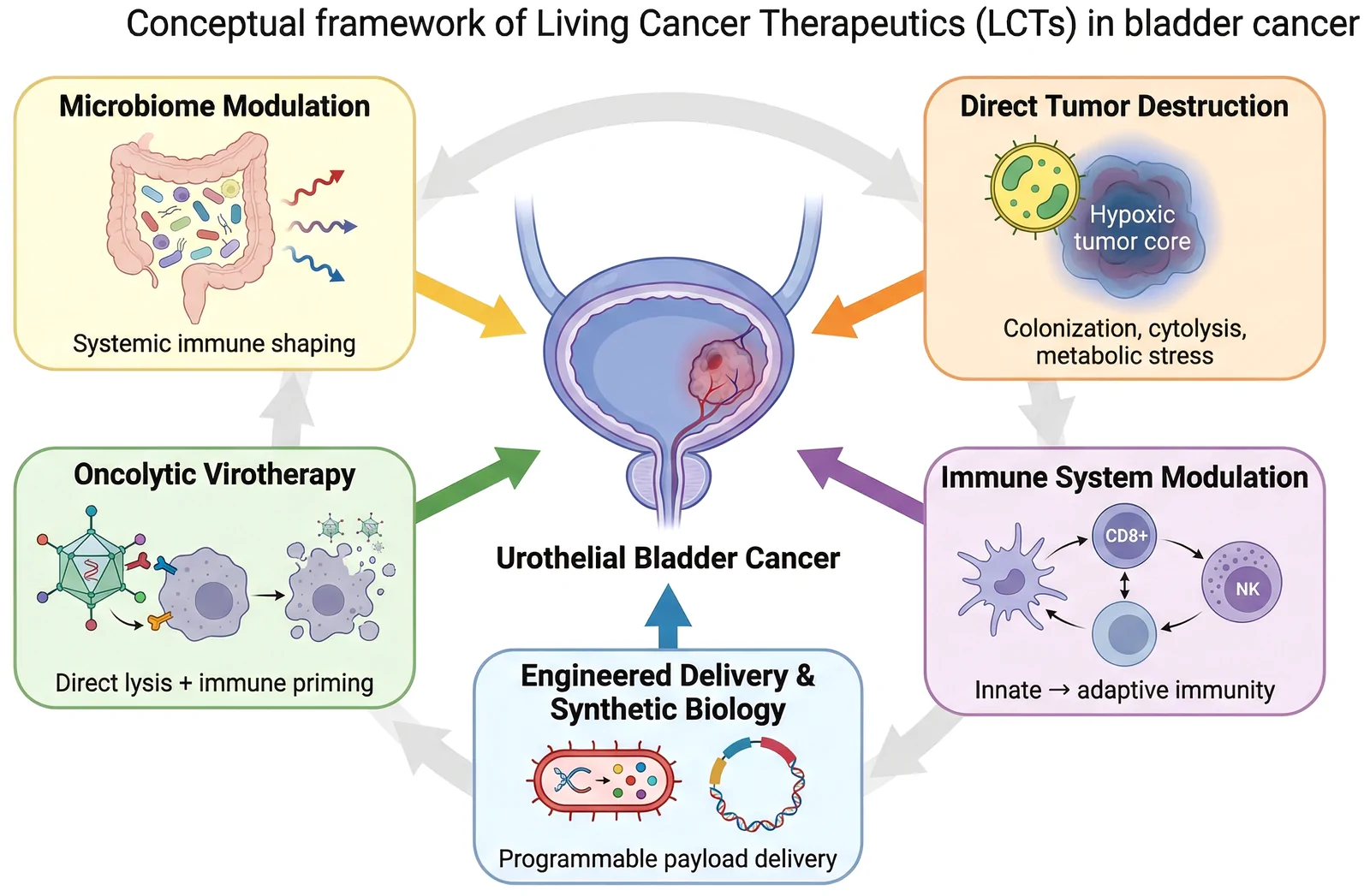
Conceptual framework of LCTs in UBC.

Unlike chemotherapy or radiotherapy, which frequently lack tumor specificity and may cause substantial systemic toxicity, LCTs—a heterogeneous group of microbial and biologically active therapeutic platforms—utilize intrinsic biological properties to selectively target tumor tissue, induce cancer cell death, and modulate the tumor microenvironment (26). In general, the mechanisms of action of LCTs can be classified into five main categories, which are schematically summarized in **Figure 3**.

### 3.1 Direct tumor destruction

Certain bacteria exert direct antitumor activity through invading tumors, competing for metabolic resources, and releasing cytotoxic factors that induce cancer cell death Species such as *Listeria monocytogenes* have been associated with reactive oxygen species (ROS) production and modulation of host immune responses, mechanisms that may contribute to antitumor activity (28), while *Escherichia coli* secretes cytolysin A, leading to membrane disruption and caspase-dependent cell death (29). In a rodent UBC model, Bifidobacterium infantis–mediated HSV-TK/ganciclovir therapy significantly inhibited tumor growth and induced the robust apoptosis. Treatment upregulated Fas/FasL, cytochrome c, and caspase-9, consistent with activation of both extrinsic and intrinsic apoptotic pathways (30). However, these findings remain limited to preclinical animal models, and clinical efficacy in humans has not yet been established.

### 3.2 Immune system modulation

A central hallmark of LCTs is their capacity to activate and shape antitumor immunity. Both bacteria and viruses function as potent immune stimulators, capable of initiating innate immune responses that subsequently prime durable adaptive immunity.

#### 3.2.1 Innate-to-adaptive immune activation

Microorganisms are potent activators of the innate immune system. Upon exposure to bacteria or viruses, host immune cells, including neutrophils, macrophages, natural killer (NK) cells, and dendritic cells, are rapidly recruited and activated, accompanied by the release of proinflammatory cytokines such as interleukin (IL)-1, IL-6, tumor necrosis factor–α (TNF-α), and IL-12 (31). Conserved microbial structures, including lipopolysaccharides (LPS) and flagellin, act as pathogen-associated molecular patterns (PAMPs) that engage toll-like receptors (TLRs) and related pattern-recognition receptors, thereby promoting monocyte–macrophage differentiation and activation and antigen-presenting cell maturation (32, 33). Beyond classical bacterial species, emerging LCTs such as *Hominenteromicrobium mulieris* and *Enterococcus gallinarum* demonstrated the ability to stimulate dendritic cells and enhance cytokine production, leading to amplified innate antitumor immunity and improved responsiveness to immunotherapies (34–36).

Preclinical studies supports the relevance of this mechanism in UBC. Seow et al. evaluated *Lactobacillus rhamnosus* GG (LGG) in an orthotopic murine UBC model, demonstrating that both intravesical and oral LGG significantly increased cure rates compared with controls and achieved outcomes comparable to BCG. LGG treatment restored XCL1 expression, a chemokine associated with antitumor immune recruitment, enhanced neutrophil and macrophage infiltration, and resulted in cure rates of 89%, versus 20% in untreated animals (37). These observations are derived primarily from murine models and require validation in human clinical studies. Clinical translation of innate-to-adaptive immune amplification has been demonstrated in BCG-unresponsive disease. In the QUILT-3.032 trial, patients with BCG-unresponsive disease received the immune cell–activating interleukin-15 (IL-15) superagonist Nogapendekin alfa inbakicept with BCG or as monotherapy. This combination signals activation and expansion of NK and CD8^+^ T cells, amplifying BCG-driven antitumor immune responses. A complete response was achieved in 62% of patients, with durable responses persisting at 12 and 24 months in many cases and 24-month cystectomy-free survival and disease-specific survival rates of 89.2% and 100%, respectively (25, 38).

#### 3.2.2 Vaccine-based and *in situ* adaptive immune responses

Bacteria-based cancer vaccines represent a complementary strategy to reinforce adaptive antitumor immunity. A related strategy is *in situ* vaccination, in which recombinant bacteria express or release tumor antigens directly within the tumor microenvironment, promoting local antigen presentation and systemic T-cell priming (39).

In BCG-unresponsive disease, intravesical administration of *Mycobacterium phlei* cell wall–nucleic acid (MCNA) demonstrated a favorable safety profile when administered perioperatively. Immediate postoperative instillation was well tolerated, with predominantly mild local adverse events, supporting the combined pro-apoptotic and immunotherapeutic activity and justifying further evaluation (40). Similarly, a phase I open-label study assessed an adjuvanted MAGE-A3 cancer vaccine combined with intravesical BCG in NMIBC patients. The combination was well tolerated and induced seroconversion and systemic vaccine-specific T-cell responses, while BCG promoted local infiltration of antigen-specific T cells within the bladder mucosa (41).

Viral-based vaccine platforms can stimulate adaptive immunity in bladder cancer. Intravesical recombinant fowlpox viruses encoding granulocyte–macrophage colony-stimulating factor (GM-CSF) or the three immune costimulatory molecules B7.1, ICAM-1, and LFA-3 (TRICOM) induced local immune cell infiltration and systemic antibody responses in BCG-unresponsive patients. Treatment was well tolerated, with transient liver enzyme elevations, supporting the safety and immunogenicity of intravesical viral vaccination approaches (42).

#### 3.2.3 BCG as archetypal oncobiotic

Bacillus Calmette–Guérin (BCG) initiates a complex, multistep immune cascade beginning with bacterial attachment to the urothelium and internalization by epithelial and antigen-presenting cells. This process triggers the recruitment of neutrophils, macrophages, dendritic cells, NK cells, and T lymphocytes, accompanied by the release of proinflammatory cytokines and chemokines including IL-2, IL-12, interferon-γ (IFN-γ), TNF, IL-1, IL-6, and GM-CSF (43–45). The broad protective effects of BCG likely arise from two complementary mechanisms: heterologous T-cell immunity and trained immunity. Trained immunity refers to enhanced, nonspecific innate immune responses induced by prior antigen exposure, providing protection against subsequent infections independently of classical B- and T-cell–mediated adaptive immunity (46).

**Figure 4:**
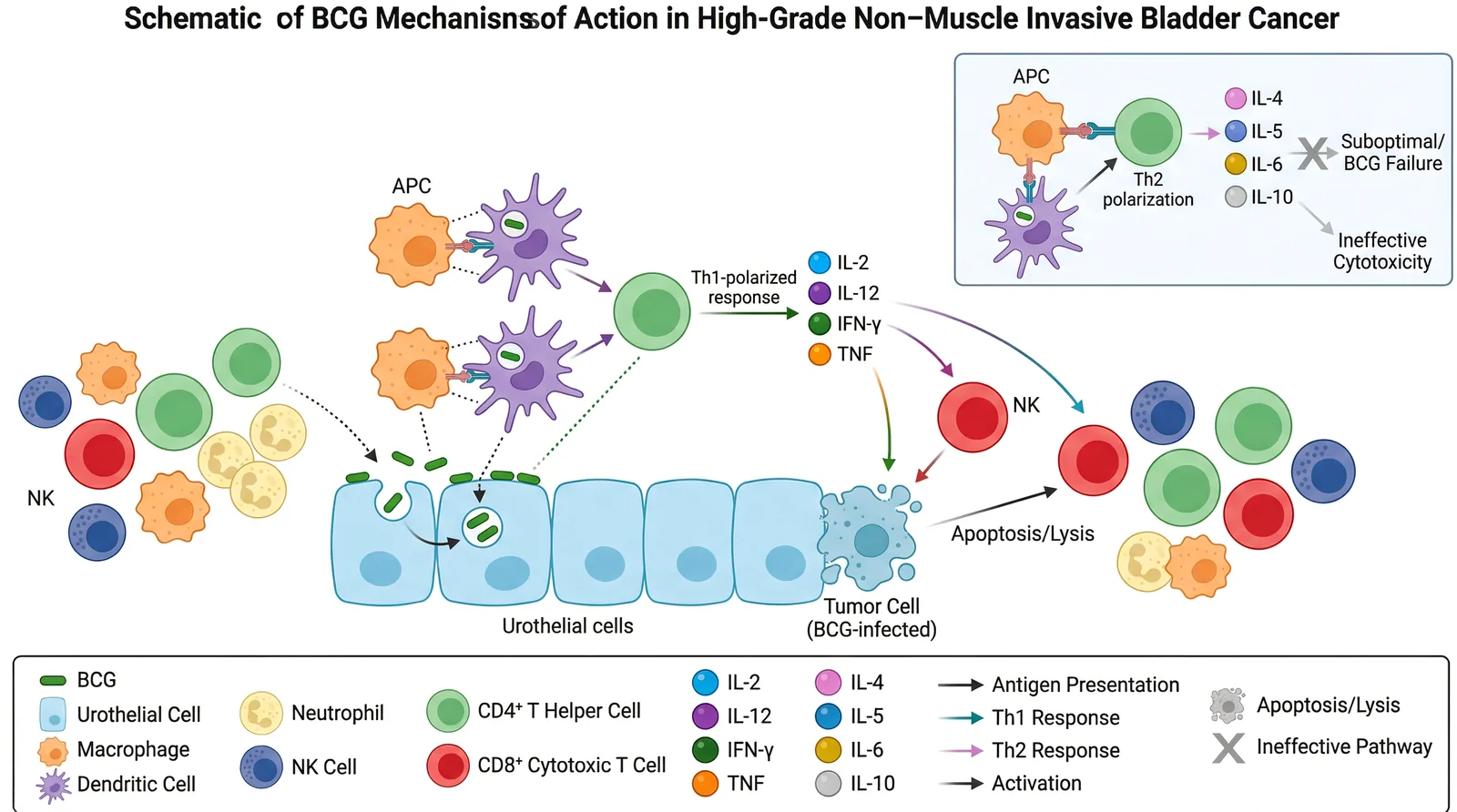
BCG mechanisms in high grade non-muscle invasive bladder cancer.

Compared with untreated patients, BCG-treated individuals show increased macrophage infiltration in urine and bladder tissue, along with enhanced cytotoxic activity against bladder cancer cells. However, elevated CD68^+^CD163^+^ (M2) macrophages are associated with recurrence, suggesting suppression of BCG-induced antitumor immunity (47). Antigen-presenting cells process BCG antigens and present them to BCG-specific CD4^+^ T cells, promoting a Th1-dominant immune response that enhances cytotoxic activity by macrophages, CD8^+^ T cells, NK cells, and other effector populations (48). Because live BCG can cause infectious complications in some patients, efforts have focused on identifying its immune-active components responsible for antitumor effects. These include PRR-activating molecules, trehalose dimycolate, immunogenic proteins such as Ag85b, γδ T-cell–recognized antigens, and the BCG cell wall skeleton (47) (**Figure 4**).

### 3.3 Drug delivery systems and synthetic engineering

Engineered bacteria have been extensively investigated as tumor-selective vectors for cytotoxic genes and immunomodulatory molecules. Engineered *Salmonella* strains have been used to deliver small interfering RNA targeting programmed cell death protein-1 (PD-1), resulting in reduced tumor growth, enhanced apoptosis, increased CD8^+^ T-cell infiltration, and improved systemic immune activation in preclinical UBC models (49).

Viral vectors also function as highly efficient delivery platforms. CV301 is a recombinant poxvirus-based cancer vaccine incorporating vaccinia, modified vaccinia Ankara–Bavarian Nordic, and fowlpox vectors. A single-arm Phase II trial evaluated CV301 in combination with atezolizumab as first-line therapy for cisplatin-ineligible patients with advanced UBC or those with disease progression after platinum-based chemotherapy. Among 43 treated patients, nine experienced treatment-related adverse events of grade ≥3. Objective clinical responses were limited, leading to early trial termination for futility; however, clinical benefit was associated with CEA- and MUC-1–specific T-cell responses (50). In contrast, nadofaragene firadenovec—a non-replicating adenovirus encoding interferon-α2b—represents a clinically successful example of viral gene delivery. In a phase III multicenter trial, intravesical administration achieved complete responses in over half of treated patients at three months, with durable responses and minimal high-grade toxicity (51).

Advances in synthetic biology further enabled the development of programmable LCTs equipped with safety “kill switches,” environmental biosensors, and tightly regulated payload-release mechanisms. These features allow precise spatial and temporal control of therapeutic activity while reducing off-target effects (52, 53). A representative example is the engineering of attenuated *Salmonella typhimurium* VNP20009 with a temperature-responsive plasmid encoding the interleukin-15 (IL-15) superagonist complex IL-15/IL-15Rα. Upon thermal activation, these bacteria secreted functional cytokine complexes, inhibited bladder tumor growth following intravesical administration, and further enhanced therapeutic efficacy when used sequentially with epirubicin, without added toxicity (54).

Synthetic viral systems have similarly been applied to localized gene therapy. Non-replicative human papillomavirus pseudovirions have been evaluated as intravesical delivery vehicles for HSV-TK–based suicide gene therapy in UBC. In orthotopic murine models, these pseudovirions selectively infected tumor cells and, in combination with ganciclovir, induced immunogenic tumor cell death, activated tumor-specific CD8^+^ T-cell responses, reduced tumor growth, and improved survival, supporting their therapeutic potential as safe and controllable delivery platforms (55). Together, these studies show that engineered microbes act as programmable delivery platforms, enabling targeted transport of therapeutic payloads to bladder tumors while improving efficacy and minimizing systemic toxicity in UBC.

### 3.4 Oncolytic viruses (direct lysis and immune priming)

Oncolytic virotherapy represents a distinct class of LCTs that exploit the natural or engineered ability of viruses to selectively infect, replicate within, and destroy malignant cells while simultaneously activating anti-tumor immune responses. Oncolytic viruses exert their therapeutic effects through preferential replication in cancer cells, direct tumor cell lysis, and induction of immunogenic cell death, resulting in the release of tumor-associated antigens and inflammatory signals that promote adaptive immune activation (14, 56). Depending on viral properties and clinical context, oncolytic viruses may be administered intravenously, intravesical, or intratumorally.

Early preclinical work established the feasibility of transcriptionally targeted oncolytic adenoviruses for bladder cancer. In 2002, Zhang et al. developed CG8840 by placing adenovirus type 5 under the control of the human uroplakin II promoter, thereby restricting viral replication to urothelial carcinoma cells. CG8840 demonstrated selective replication in malignant urothelial cells and significantly inhibited tumor growth in human bladder cancer xenograft models after intravenous or intratumoral administration (57). Although CG8840 itself was an early experimental construct, it provided important proof-of-concept for the later development of clinically advanced bladder cancer -specific oncolytic adenoviruses such as cretostimogene grenadenorepvec (CG0070). Subsequent studies further refined adenoviral selectivity. Lichtenegger et al. demonstrated that XVir-N-31, a YB-1–dependent oncolytic adenovirus, induced enhanced immunogenic cell death and superior tumor growth inhibition compared with wild-type adenovirus in UBC models (58).

Translation into early clinical evaluation has supported the safety and biological activity of intravesical oncolytic virotherapy. Annels et al. conducted a phase I study assessing intravesical administration of bioselected coxsackievirus A21 (CVA21; CAVATAK), alone or combined with mitomycin C, in treatment-naïve patients with NMIBC prior to transurethral resection. Treatment was well tolerated and associated with intratumoral viral replication, tumor inflammation, immune gene upregulation, and a complete response in one patient, supporting further clinical development of CVA21 in NMIBC (59).

Among oncolytic viruses evaluated in UBC, coxsackievirus-adenovirus receptor (CAR)–targeting and immune-armed adenoviruses have shown particularly promising results. CG0070 is a genetically modified adenovirus designed to selectively replicate in retinoblastoma (Rb) pathway–deficient UBC cells. Viral replication is driven by the E2F-1 promoter, while expression of granulocyte–macrophage colony-stimulating factor (GM-CSF) enhances local immune activation. Phase I evaluation of intravesical CG0070 demonstrated a favorable safety profile and complete responses in nearly half of treated patients, with higher response rates observed in multidose cohorts (60). In the subsequent BOND-2 phase II trial, CG0070 achieved a six-month complete response rate of 47% in patients with BCG-unresponsive NMIBC, with particularly favorable outcomes in CIS–containing tumors and minimal disease progression (61). Combination strategies have further enhanced efficacy; in the CORE-001 phase II trial, CG0070 administered with pembrolizumab resulted in durable complete responses in over half of treated patients at 12 months, with no progression to muscle-invasive disease (62).

Additional viral platforms have expanded the scope of oncolytic virotherapy in UBC. Vesicular stomatitis virus (VSV) variants have demonstrated selective activity in interferon-nonresponsive tumors. Hadaschik et al. showed that both wild-type VSV and the mutant d51M variant (AV3) preferentially lysed aggressive UBC cell lines and, when administered intravesical, reduced tumor burden by up to 98% in murine models (63). Similarly, an F4L-deleted oncolytic vaccinia virus exploiting elevated ribonucleotide reductase M2 (RRM2) expression in tumor cells exhibited selective replication, potent tumor regression, and induction of antitumor immunity in both rat and human bladder cancer models, without toxicity to normal tissue (64).

Beyond adenoviruses and VSV, other oncolytic viruses have demonstrated immunomodulatory potential. Intravesical administration of MV-NIS, an oncolytic measles virus encoding the sodium-iodide symporter, was evaluated in patients undergoing cystectomy and resulted in tumor downstaging and marked immune cell infiltration with minimal toxicity (65, 66). OH2, a genetically engineered herpes simplex virus type 2 expressing GM-CSF, combines direct oncolysis with immune stimulation and is currently under clinical evaluation in patients with NMIBC unresponsive to first-line intravesical therapy (67, 68). Newcastle disease virus (NDV) has also been shown to induce immunogenic cell death, upregulate major histocompatibility complex and PD-L1 expression, and enhance the efficacy of immune checkpoint inhibitors when administered intratumorally in bladder cancer models (69).

Comparative studies further underscore the immunological advantages of oncolytic virotherapy. In preclinical UBC models, VSVd51-GM-CSF elicited stronger immunogenic cell death, cytokine release, immune cell infiltration, and survival benefits than BCG, including in BCG-resistant disease. These effects were recapitulated in patient-derived organoids, supporting the translational relevance of oncolytic virus-mediated immune priming in NMIBC (70).

**Figure 5:**
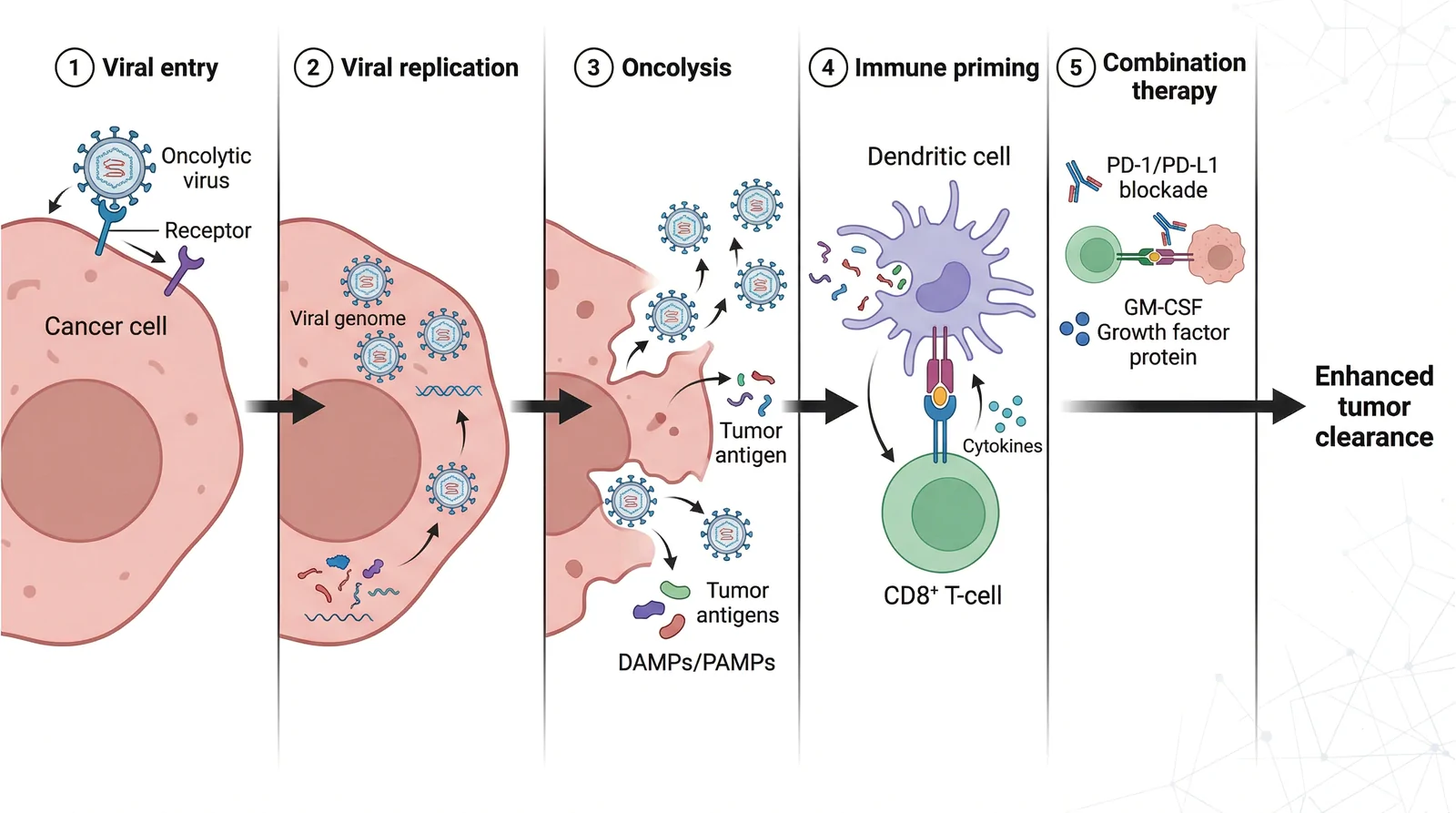
Antitumor mechanisms of oncolytic virotherapy.

The clinical relevance of intravesical CVA21 was further demonstrated in the phase I CANON study, in which 15 patients with NMIBC received CVA21 prior to surgery. Treatment, administered alone or with low-dose mitomycin C, induced tumor inflammation and immune-related gene expression, with one complete response observed, while maintaining a favorable safety profile. These findings provided early clinical proof-of-concept for CVA21–mediated immune priming in NMIBC (71). The antitumor mechanisms of oncolytic virotherapy are illustrated in **Figure 5**.

### 3.5 Microbiome & probiotic-based oncobiotics

The host microbiome plays a central role in regulating local and systemic immune responses and has emerged as an important modulator of cancer development and treatment efficacy. Although the influence of the microbiome on carcinogenesis is increasingly recognized, its specific role in UBC remains incompletely defined. Comparative 16S sequencing studies of urinary microbiomes from UBC patients and healthy controls have demonstrated similar overall microbial diversity but distinct taxonomic differences, including enrichment of *Fusobacterium* in UBC-associated samples, suggesting a potential link to tumor biology (72).

Growing evidence indicates that urinary microbiome dysbiosis contributes to chronic urothelial inflammation, immune modulation, and UBC pathogenesis. Distinct microbial profiles have been associated with altered cytokine signaling, differential responsiveness to BCG immunotherapy, and potentially immune checkpoint inhibition, suggesting that microbiome modulation may represent a future investigational strategy to improve disease prevention, prognosis, and therapeutic response. However, most currently available microbiome data in UBC remain largely associative, and causal relationships have not yet been definitively established (73). Metagenomic analyses further reveal that the urinary tract harbors a diverse microbiome—even in culture-negative urine—dominated by genera such as *Lactobacillus*, *Corynebacterium*, and *Streptococcus*. While their pathogenic or oncogenic roles remain unclear, these microbes may influence tumor behavior and treatment outcomes (74).

Clinical support for probiotic-based LCTs in UBC is provided by a trial evaluating oral *Lactobacillus casei* as an adjunct to intravesical epirubicin following transurethral resection. Patients receiving combined therapy exhibited significantly improved three-year recurrence-free survival without increased toxicity, although progression-free and overall survival were unchanged, indicating a safe enhancement of local disease control (75).

Beyond *L. casei*, a range of probiotic strains has been investigated for anticancer potential in preclinical and early clinical settings. These organisms are generally well tolerated and offer favorable safety profiles; however, their standalone antitumor efficacy is limited, and complete tumor eradication is rarely achieved. Consequently, current research efforts are focused on optimizing probiotic-based strategies through improved delivery systems, genetic engineering, and rational combination with established oncologic therapies (26, 76, 77). Such approaches aim to amplify immunostimulatory effects while preserving the inherent safety advantages of probiotic platforms.

**Figure 6:**
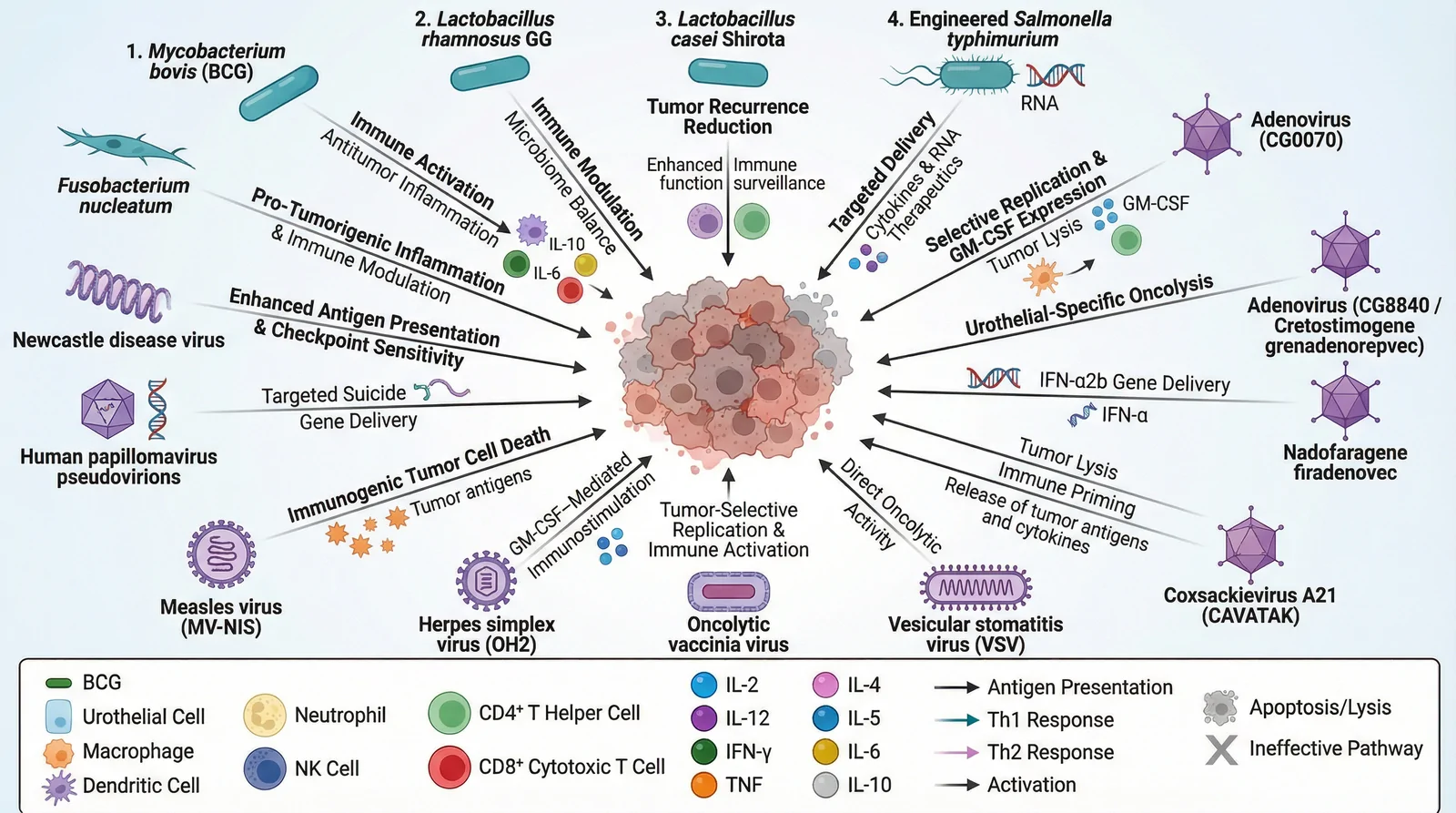
Examples of targeted microbial and viral therapeutics in bladder cancer.

Advances in bioengineering have further expanded the microbiome-based oncobiotic landscape through the development of bacteria-based microrobots, or bacteriobots. These engineered bacterial systems are designed to selectively target tumor tissue and either deliver therapeutic agents or mediate localized tumor disruption. A multifunctional microrobot using magnetically guided, thermally responsive *E. coli* Nissle 1917 enabled collective tumor targeting, real-time fluorescence tracking, and effective cancer cell killing through combined magnetothermal ablation and ROS-mediated apoptosis *in vitro* and *in vivo* (78). Although still in early developmental stages, bacteriobots represent a promising next-generation extension of microbiome-informed LCTs. Examples of targeted microbial and viral therapeutics in bladder cancer are summarized in **Figure 6**.

## 4 Discussion

This narrative review synthesizes current evidence on LCTs in UBC and highlights how bacterial, viral, and microbiome-based LCTs exert antitumor effects through five interconnected mechanisms: direct tumor destruction, immune modulation, engineered drug delivery, oncolytic virotherapy, and microbiome-driven immune regulation. Although presented as discrete categories, these mechanisms frequently converge on immune activation, which appears to be the central determinant of durable clinical benefit. Furthermore, ongoing and emerging clinical trials investigating LCTs and oncolytic platforms for UBC are summarized in **Table 2**.

**Table 2:** Ongoing and emerging clinical trials of living cancer therapeutics and oncolytic platforms in bladder cancer.

Clinical trial identifier	Therapeutic platform	Treatment category	Study status	Disease setting	Intervention / strategy	Main objective
NCT06668493	Nadofaragene firadenovec	Recombinant adenoviral IFN-α gene therapy	Recruiting	Low-grade UTUC	Administration of nadofaragene firadenovec to the renal pelvis	Assessment of safety and efficacy in upper tract urothelial disease
NCT07332351 (TRIFECTA)	Nadofaragene firadenovec + gemcitabine/cisplatin + durvalumab	Combination gene-immunochemotherapy	Not yet recruiting	MIBC	Neoadjuvant intravesical nadofaragene firadenovec combined with systemic chemotherapy and immune checkpoint inhibition	Evaluation of bladder-preserving multimodal neoadjuvant therapy
NCT06510374	Nadofaragene firadenovec	Recombinant adenoviral IFN-α gene therapy	Recruiting	Intermediate-risk NMIBC	Intravesical nadofaragene firadenovec versus observation	Prevention of recurrence and disease progression
NCT06545955	Nadofaragene firadenovec ± chemotherapy/immunotherapy	Combination intravesical gene therapy	Recruiting	BCG-unresponsive NMIBC	Intravesical nadofaragene firadenovec alone or combined with chemotherapy or immunotherapy	Evaluation of enhanced antitumor efficacy and response durability
NCT06253845	CG0070	Oncolytic adenoviral therapy	Active, not recruiting	Intermediate-risk NMIBC	Intravesical CG0070 following TURBT	Reduction of post-resection recurrence
NCT04452591 (BOND-003)	Cretostimogene grenadenorepvec (CG0070)	Oncolytic adenoviral therapy	Active, not recruiting	High-risk BCG-unresponsive NMIBC	Intravesical cretostimogene grenadenorepvec after BCG failure	Evaluation of complete response durability and bladder preservation
NCT05232136	OH2	Oncolytic HSV-2 therapy	Recruiting	NMIBC	Intravesical OH2 oncolytic viral therapy	Preliminary evaluation of intravesical oncolytic activity
NCT06427291	T3011	Oncolytic viral therapy	Recruiting	NMIBC	Intravesical instillation of T3011	Evaluation of intravesical viral therapy efficacy
NCT07359235	KD01	Recombinant/oncolytic adenoviral therapy	Recruiting	Bladder cancer	Intravesical administration of KD01	Early clinical assessment of intravesical immunotherapy
NCT06632964	VT-101	Recombinant/oncolytic adenoviral therapy	Recruiting	NMIBC	VT-101 investigational therapy	Investigation of therapeutic activity in NMIBC
NCT06181266	ZH9	Salmonella enterica serovar Typhi–based bacterial immunotherapy	Active, not recruiting	High-risk NMIBC	Intravesical ZH9 treatment	Phase 1/1b assessment of dosing, safety, and preliminary efficacy
NCT06833073 (V940-011 / INTerpath-011)	Intismeran autogene (V940) + BCG	Personalized mRNA immunotherapy	Recruiting	High-risk NMIBC with CIS	Personalized neoantigen mRNA immunotherapy combined with BCG	Evaluation of recurrence prevention and immune response enhancement

(Access 1 May 2026).

Direct tumor destruction through bacterial colonization and cytotoxicity remains a potent yet underutilized strategy in UBC. Facultative and obligate anaerobes preferentially localize to hypoxic and necrotic tumor regions that are poorly accessible to conventional therapies. Within these niches, bacteria can suppress tumor growth through toxin secretion, enzymatic degradation, nutrient depletion, and metabolic competition. While safety concerns have historically limited clinical translation, advances in genetic attenuation, auxotrophy, hypoxia-responsive promoters, and inducible kill switches have substantially improved controllability, suggesting renewed translational potential for cytolytic bacterial therapies (27, 79).

Immune modulation represents the most clinically mature and validated LCT mechanism. The long-standing success of BCG therapy in NMIBC exemplifies the capacity of LCTs to convert local innate immune activation into durable adaptive antitumor responses (11). Emerging bacterial and viral platforms appear capable of recapitulating—and in some settings exceeding—BCG-mediated immune activation, particularly in BCG-unresponsive disease. These findings underscore the suitability of UBC for immune-driven local therapies, given its anatomical accessibility, established intravesical treatment paradigm, and relatively localized disease course.

Engineered drug delivery and synthetic biology approaches further unify oncobiotic strategies by repurposing bacteria and viruses as programmable therapeutic carriers. Delivery of suicide genes, cytokines, immune modulators, and nucleic acids directly to the tumor microenvironment has improved efficacy while minimizing systemic toxicity (49, 54, 78, 80). Clinical success with nadofaragene firadenovec highlights the translational feasibility of this approach, particularly for patients in whom systemic therapies are poorly tolerated (51). Advances such as inducible promoters, biosensors, and safety switches are especially advantageous in UBC, where intravesical administration allows precise spatiotemporal control.

Oncolytic virotherapy represents one of the most advanced LCT modalities, integrating direct tumor lysis with immune priming. Across diverse viral families, oncolytic platforms consistently induce immunogenic cell death and enhance antitumor immunity, with immune-armed viruses demonstrating synergy with immune checkpoint inhibition (10, 14, 81, 82). Nonetheless, challenges such as antiviral immunity, receptor heterogeneity, and optimal treatment sequencing remain important barriers to broader adoption. Long-term safety monitoring remains essential, particularly for replicating or genetically modified organisms. Although advances in genetic engineering have improved tumor selectivity and controllability, concerns regarding unintended dissemination, immune-related toxicity, and long-term biological effects remain important considerations for future clinical implementation.

Beyond biological safety, practical translational barriers must also be addressed. Manufacturing scalability, product standardization, storage stability, and regulatory oversight remain substantial barriers to widespread clinical implementation of living biologic therapies. These challenges are particularly relevant for personalized or genetically engineered platforms, where reproducibility, quality control, and cost-effectiveness may significantly influence clinical adoption.

Microbiome- and probiotic-based LCTs represent a more indirect but increasingly influential mechanism of LCT action. While native probiotics exhibit limited standalone antitumor activity, engineered strains and bacteria-based microrobots offer opportunities to integrate immune modulation with targeted delivery and precision control. However, the causal relationship between urinary microbiome alterations and tumor biology remains incompletely defined, necessitating longitudinal and mechanistic studies.

Several overarching principles emerge. First, combination strategies consistently outperform monotherapies, reflecting the multifactorial nature of tumor immune evasion. Second, immune activation underlies durable benefit across LCT modalities, even when primary mechanisms involve cytotoxicity or gene delivery. Third, UBC provides unique advantages for LCT development due to its accessibility and suitability for localized therapy.

Despite substantial progress, important limitations remain within the current evidence base for LCTs in UBC. Most available data originate from preclinical studies and early-phase clinical trials with heterogeneous methodologies, small patient populations, and variable endpoints, limiting direct comparison and definitive conclusions regarding comparative efficacy. Several biological and translational barriers also remain unresolved.

Pre-existing or treatment-induced antiviral neutralizing antibodies may reduce the efficacy of oncolytic viral therapies by limiting viral persistence and tumor penetration. In addition, intravesical delivery may be hindered by urothelial barriers, urine dilution, and tumor heterogeneity, potentially resulting in inconsistent therapeutic exposure. Host immune responses may further compromise treatment efficacy through premature clearance of therapeutic microorganisms or excessive inflammatory reactions.

Safety concerns related to microbial or viral shedding, unintended dissemination, and long-term biosafety require continued evaluation, particularly for genetically engineered or replicating platforms. Furthermore, multiple approaches demonstrating promising preclinical activity have historically failed to achieve durable clinical benefit in humans. Practical challenges including complex GMP manufacturing requirements, regulatory oversight, high development costs, and difficulties with product standardization and reproducibility may further restrict broader clinical implementation.

In addition, currently available clinical studies are generally limited by short follow-up duration, restricting assessment of long-term efficacy, recurrence prevention, durability of response, and delayed adverse events. Consequently, predictive biomarkers, long-term safety monitoring, and large-scale randomized clinical trials remain essential before widespread integration of LCTs into routine bladder cancer management.

## 5 Clinical implications and future research

Living cancer therapeutics (LCTs) represent a promising shift in the management of urinary bladder cancer (UBC), particularly in the context of rising treatment costs, aging patient populations, and high recurrence rates. Clinically, LCTs offer the potential for bladder-preserving strategies that combine tumor selectivity with immune activation while minimizing systemic toxicity. Intravesical administration provides a unique advantage in UBC, enabling high local drug concentrations and controlled exposure. Emerging therapies such as gene-modified adenoviruses and IL-15–based immune agonists demonstrate that engineered biologics can achieve durable responses in BCG-unresponsive disease, a population with limited options.

Future research should prioritize biomarker-driven patient selection, as immune polarization, tumor molecular subtype, and microbiome composition may influence therapeutic response. Rational combination strategies integrating LCTs with immune checkpoint inhibitors, chemotherapy, or radiotherapy warrant systematic evaluation in well-designed randomized trials. Long-term safety monitoring remains essential, particularly for replicating or genetically modified organisms. Advances in synthetic biology, precision delivery systems, and microbiome engineering may further enhance specificity and controllability. Ultimately, multidisciplinary collaboration will be crucial to translate LCT innovation into standardized, cost-effective clinical practice.

## 6 Conclusion

In summary, this review highlights LCTs as a multifaceted and rapidly evolving therapeutic paradigm in UBC. By integrating direct tumor targeting, immune modulation, programmable delivery systems, oncolytic virotherapy, and microbiome-informed strategies, oncobiotics offer a unique opportunity to enhance therapeutic efficacy while minimizing toxicity. Continued mechanistic insight, rigorous clinical evaluation, and thoughtful integration with existing treatment modalities will be essential to unlock their full potential. As evidence matures, LCTs may contribute to the future evolution of bladder-preserving therapeutic strategies, particularly for patients with BCG-unresponsive disease and those seeking bladder-preserving alternatives.
