# Correction: Oncobiotics in urinary bladder cancer. A narrative review of living cancer therapeutics

**DOI:** 10.3389/fonc.2026.1951323

**Published:** 2026-09-10

**Authors:** Frontiers Production Office

**Affiliations:** Frontiers Media SA, Lausanne, Switzerland

**Keywords:** bacteria, bladder cancer, immunotherapy, living cancer therapeutics, oncobiotics, oncolytic viruses, probiotics

Due to a production error, there were some mistakes in the published version, which have been corrected and are described below.

The first sentence was rephrased for clarity. A correction has been made to the section **Introduction:**

“Urinary bladder cancer (UBC) was among the most prevalent cancers worldwide, ranking seventh by five-year prevalence in 2020, with more than 1.7 million people living within five years of diagnosis.”

A sentence was rephrased for clarify. A correction has been made to the section 3 **LCT-mediated mechanisms of oncobiotics in UBC-oncotherapy**, 3.1 *Direct tumor destruction:*

“Species such as *Listeria monocytogenes* have been associated with reactive oxygen species (ROS) production and modulation of host immune responses, mechanisms that may contribute to antitumor activity (27), while *Escherichia coli* secretes cytolysin A, leading to membrane disruption and caspase-dependent cell death (28).”

A correction has been made to Section 3.4 *Oncolytic viruses (direct lysis and immune priming)*, Paragraph 2:

“Early preclinical work established the feasibility of transcriptionally targeted oncolytic adenoviruses for bladder cancer. In 2002, Zhang et al. developed CG8840 by placing adenovirus type 5 under the control of the human uroplakin II promoter, thereby restricting viral replication to urothelial carcinoma cells. CG8840 demonstrated selective replication in malignant urothelial cells and significantly inhibited tumor growth in human bladder cancer xenograft models after intravenous or intratumoral administration (55). Although CG8840 itself was an early experimental construct, it provided important proof-of-concept for the later development of clinically advanced bladder cancer–specific oncolytic adenoviruses such as cretostimogene grenadenorepvec (CG0070).”

Sievert, K.D., Amend, B., Nagele, U. et al. Economic aspects of bladder cancer: what are the benefits and costs?. World J Urol 27, 295–300 (2009). https://doi.org/10.1007/s00345-009-0395-z was not cited in the article. The citation has now been inserted in the section Introduction, Paragraph 1, after the sentence:

“UBC is recognized as the cancer entailing the highest lifetime treatment costs per patient.”

Lin NYT, Fukuoka S, Koyama S, et al. Microbiota-driven antitumour immunity mediated by dendritic cell migration. Nature. (2025) 644:1058–1068. doi: 10.1038/s41586-025-09249–8 was not cited in the article. The citation has now been inserted in the section 3.2.1 *Innate-to-adaptive immune activation* after the sentence:

“Beyond classical bacterial species, emerging LCTs such as Hominenteromicrobium mulieris and Enterococcus gallinarum demonstrated the ability to stimulate dendritic cells and enhance cytokine production, leading to amplified innate antitumor immunity and improved responsiveness to immunotherapies”

Miest T, Leibovich B, Bardot S, Young P, Boorjian S, Gonzalgo M, et al. 422 Safety and efficacy of neoadjuvant intravesical oncolytic MV-NIS in patients with urothelial carcinoma. *J Immunother Cancer. (2021) 9. doi: 10.1136/jitc-2021-SITC2021.422*

was not cited in the article. The citation has now been inserted in Section 3.4 *Oncolytic viruses (direct lysis and immune priming)*, after the sentence:

“Beyond adenoviruses and VSV, other oncolytic viruses have demonstrated immunomodulatory potential. Intravesical administration of MV-NIS, an oncolytic measles virus encoding the sodium-iodide symporter, was evaluated in patients undergoing cystectomy and resulted in tumor downstaging and marked immune cell infiltration with minimal toxicity.”

Binhui Biopharmaceutical Co., Ltd. OH2 Oncolytic Viral *Therapy in Non-Muscle-Invasive Bladder Cancer*. ClinicalTrials.gov identifier: NCT05232136. Bethesda (MD): National Library of Medicine (US); updated August 12, 2024. Available from: https://clinicaltrials.gov/study/NCT05232136 was not cited in the article. The citation has now been inserted in 3.4 *Oncolytic viruses (direct lysis and immune priming)*, after the sentence:

“OH2, a genetically engineered herpes simplex virus type 2 expressing GM-CSF, combines direct oncolysis with immune stimulation and is currently under clinical evaluation in patients with NMIBC unresponsive to first-line intravesical therapy.”

There was a mistake in **Table 1** as published. In the “*Developmental stage” column, the entry for CVA21* (CAVATAK) was erroneously given as “Phase I/II”. The correct developmental stage is “Phase I”. The corrected **Table 1** appears below.

**Table 1:** Overview of living cancer therapeutics in urinary bladder cancer.

Therapeutic platform	Type of LCT	Mechanism of action	Route of administration	Immune effects	Developmental stage	Translational/clinical status
BCG (Bacillus Calmette–Guérin)	Live attenuated bacteria	Activation of innate and adaptive antitumor immunity	Intravesical	Strong activation of macrophages, NK cells, dendritic cells, and T cells	Clinically established	Current standard of care for high-risk NMIBC
CG0070	Oncolytic adenovirus expressing GM-CSF	Selective replication in Rb-deficient tumor cells with GM-CSF-mediated immune activation	Intravesical	Promotes immunogenic cell death and local immune stimulation	Phase II/III	Promising efficacy in BCG-unresponsive NMIBC
CVA21 (CAVATAK)	Oncolytic coxsackievirus	Selective viral replication and tumor lysis via ICAM-1 targeting	Intravesical	Induces tumor inflammation and immune gene upregulation	Phase I	Early clinical proof-of-concept established
Nadofaragene firadenovec	Adenoviral gene therapy	Delivery of IFN-α2b gene to urothelial cells	Intravesical	Enhances interferon-mediated antitumor immunity	FDA-approved	Approved for BCG-unresponsive NMIBC
OH2	Genetically modified HSV-2	Direct oncolysis combined with GM-CSF-mediated immune activation	Intratumoral / intravesical	Enhances immune cell recruitment and activation	Phase I/II	Under clinical evaluation
MV-NIS	Oncolytic measles virus	Selective viral infection and tumor destruction	Intravesical / intravenous	Promotes immune infiltration and tumor inflammation	Early clinical development	Under clinical evaluation
Lactobacillus spp. (LGG, L. casei)	Probiotic bacteria	Immune modulation and enhancement of antitumor immune signaling	Oral and intravesical	Enhances macrophage and neutrophil recruitment	Preclinical and clinical adjunct studies	Potential adjunctive immunomodulatory strategy
Engineered Salmonella spp.	Genetically modified bacterial vector	Tumor-selective delivery of siRNA and immunomodulatory payloads	Intravesical / experimental systemic administration	Increases CD8+ T-cell infiltration and immune activation	Preclinical	Promising programmable delivery platform
Bifidobacterium infantis–HSV-TK system	Engineered probiotic suicide-gene therapy	HSV-TK/ganciclovir-mediated induction of tumor apoptosis	Experimental intravesical delivery	Indirect immune activation secondary to tumor apoptosis	Preclinical	Proof-of-concept demonstrated in animal models
IL-15–engineered Salmonella VNP20009	Synthetic bacterial immunotherapy	Localized IL-15/IL-15Rα cytokine delivery	Intravesical	Strong activation of NK cells and CD8+ T cells	Preclinical	High translational potential for immune enhancement
MCNA (Mycobacterium phlei cell wall–nucleic acid complex)	Bacterial-derived immunotherapy	Immune stimulation and pro-apoptotic activity	Intravesical	Enhances local immune activation	Early clinical evaluation	Investigated in BCG-unresponsive NMIBC
Bacteriobots (engineered E. coli Nissle 1917)	Bioengineered bacterial microrobots	Targeted tumor localization and ROS-mediated tumor destruction	Experimental local delivery	Potential secondary immune activation	Early preclinical	Emerging next-generation precision platform

There was a mistake in **Table 2** as published. In the “Treatment category” column, the following entries were erroneously given and have been corrected:

- KD01 (NCT07359235): “Intravesical viral immunotherapy” has been corrected to “Recombinant/oncolytic adenoviral therapy”.
- VT-101 (NCT06632964): “Viral immunotherapy” has been corrected to “Recombinant/oncolytic adenoviral therapy”.
- ZH9 (NCT06181266): “Viral immunotherapy” has been corrected to “Salmonella enterica serovar Typhi–based bacterial immunotherapy”.

**Table 2:** Ongoing and emerging clinical trials of living cancer therapeutics and oncolytic platforms in bladder cancer.

Clinical trial identifier	Therapeutic platform	Treatment category	Study status	Disease setting	Intervention / strategy	Main objective
NCT06668493	Nadofaragene firadenovec	Recombinant adenoviral IFN-α gene therapy	Recruiting	Low-grade UTUC	Administration of nadofaragene firadenovec to the renal pelvis	Assessment of safety and efficacy in upper tract urothelial disease
NCT07332351 (TRIFECTA)	Nadofaragene firadenovec + gemcitabine/cisplatin + durvalumab	Combination gene-immunochemotherapy	Not yet recruiting	MIBC	Neoadjuvant intravesical nadofaragene firadenovec combined with systemic chemotherapy and immune checkpoint inhibition	Evaluation of bladder-preserving multimodal neoadjuvant therapy
NCT06510374	Nadofaragene firadenovec	Recombinant adenoviral IFN-α gene therapy	Recruiting	Intermediate-risk NMIBC	Intravesical nadofaragene firadenovec versus observation	Prevention of recurrence and disease progression
NCT06545955	Nadofaragene firadenovec ± chemotherapy/immunotherapy	Combination intravesical gene therapy	Recruiting	BCG-unresponsive NMIBC	Intravesical nadofaragene firadenovec alone or combined with chemotherapy or immunotherapy	Evaluation of enhanced antitumor efficacy and response durability
NCT06253845	CG0070	Oncolytic adenoviral therapy	Active, not recruiting	Intermediate-risk NMIBC	Intravesical CG0070 following TURBT	Reduction of post-resection recurrence
NCT04452591 (BOND-003)	Cretostimogene grenadenorepvec (CG0070)	Oncolytic adenoviral therapy	Active, not recruiting	High-risk BCG-unresponsive NMIBC	Intravesical cretostimogene grenadenorepvec after BCG failure	Evaluation of complete response durability and bladder preservation
NCT05232136	OH2	Oncolytic HSV-2 therapy	Recruiting	NMIBC	Intravesical OH2 oncolytic viral therapy	Preliminary evaluation of intravesical oncolytic activity
NCT06427291	T3011	Oncolytic viral therapy	Recruiting	NMIBC	Intravesical instillation of T3011	Evaluation of intravesical viral therapy efficacy
NCT07359235	KD01	Recombinant/oncolytic adenoviral therapy	Recruiting	Bladder cancer	Intravesical administration of KD01	Early clinical assessment of intravesical immunotherapy
NCT06632964	VT-101	Recombinant/oncolytic adenoviral therapy	Recruiting	NMIBC	VT-101 investigational therapy	Investigation of therapeutic activity in NMIBC
NCT06181266	ZH9	Salmonella enterica serovar Typhi–based bacterial immunotherapy	Active, not recruiting	High-risk NMIBC	Intravesical ZH9 treatment	Phase 1/1b assessment of dosing, safety, and preliminary efficacy
NCT06833073 (V940-011 / INTerpath-011)	Intismeran autogene (V940) + BCG	Personalized mRNA immunotherapy	Recruiting	High-risk NMIBC with CIS	Personalized neoantigen mRNA immunotherapy combined with BCG	Evaluation of recurrence prevention and immune response enhancement

(Access 1 May 2026).

The corrected **Table 2** appears below.

There was a mistake in **Figure 4** as published. The title within the schematic contained a typographical error and was given as “Schematic of BCG Mechanisnssof Action in High-Grade Non–Muscle Invasive Bladder Cancer”. The title has been corrected to read “Schematic of BCG Mechanisms of Action in High-Grade Non–Muscle Invasive Bladder Cancer”. The corrected **Figure 4** appears below.

There was a mistake in the caption of **Figure 1** as published. The publication-year range was erroneously given as “(2010 - 2025)”. The corrected caption of **Figure 1** appears below:

**Figure 1:**
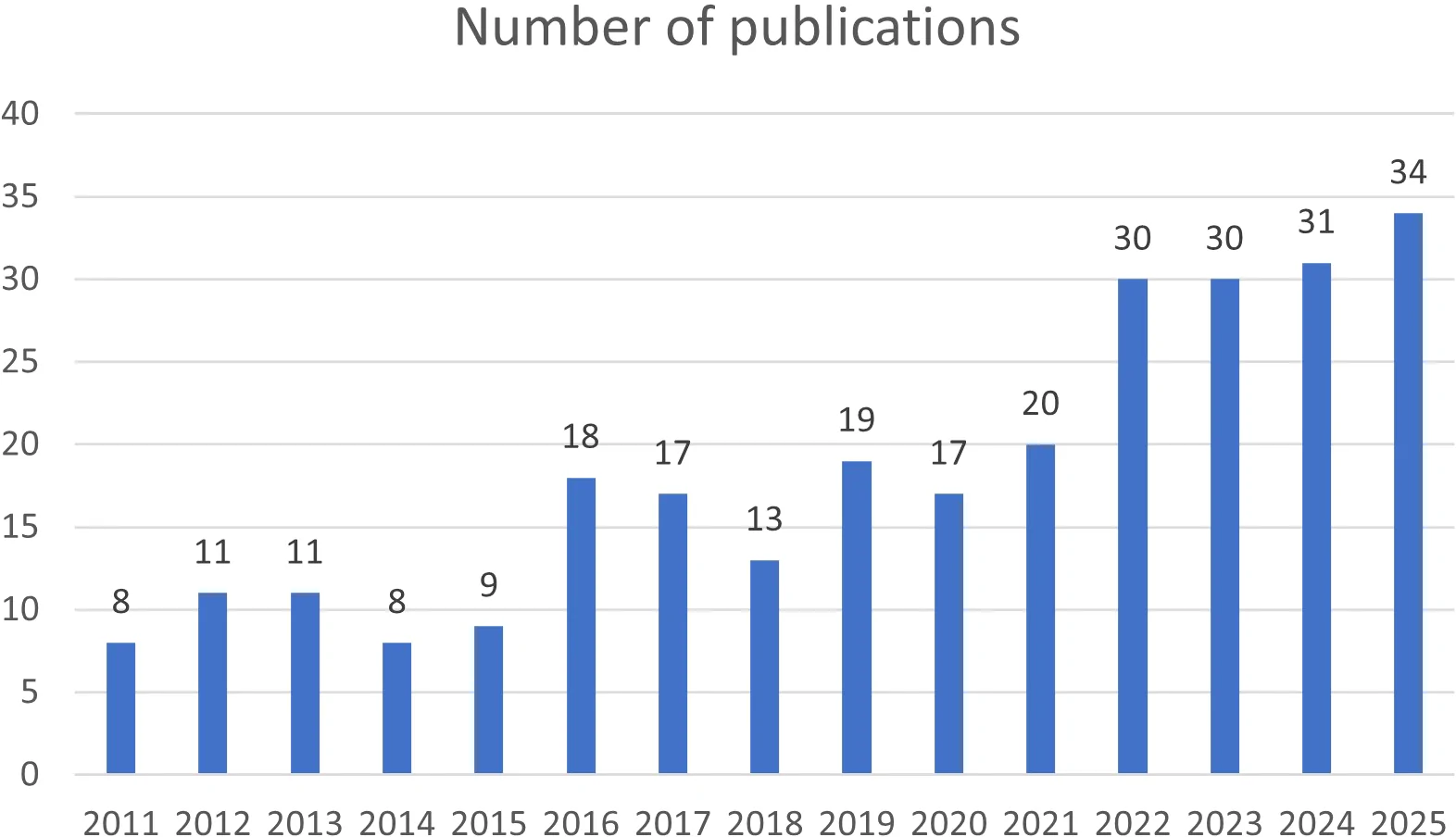
Number of publications using living cancer therapeutic with different types of oncobiotics of UBC from the last 15 years (2011-2025). Publication numbers were calculated using the PubMed database (2026, Jan 1^st^) using the keywords (oncobiotic OR "living cancer therapeutic" OR "live cancer therapeutic" OR "living therapeutic" OR "live biotherapeutic product" OR "oncolytic virus" OR virotherapy OR bacteriotherapy OR "bacterial therapy" OR "immune-modulating bacteria" ) AND ( "urinary bladder cancer"[MeSH] OR "bladder cancer" OR "bladder carcinoma" OR "urothelial carcinoma").

**Figure 4:**
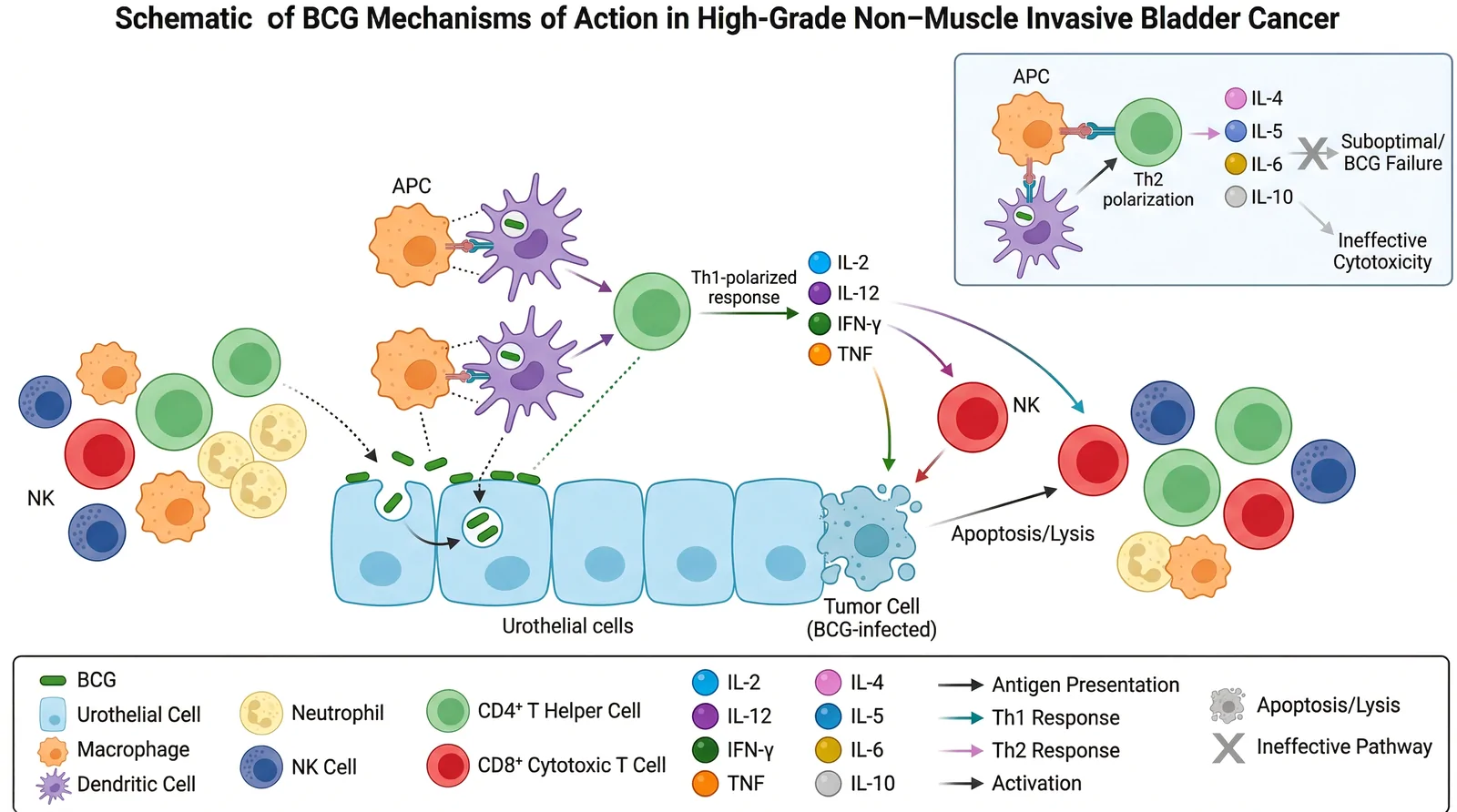
BCG mechanisms in high grade non-muscle invasive bladder cancer.

“Number of publications using living cancer therapeutic with different types of oncobiotics of UBC from the last 15 years (2011–2025). Publication numbers were calculated using the PubMed database (2026, Jan 1st) using the keywords (oncobiotic OR “living cancer therapeutic” OR “live cancer therapeutic” OR “living therapeutic” OR “live biotherapeutic product” OR “oncolytic virus” OR virotherapy OR bacteriotherapy OR “bacterial therapy” OR “immune-modulating bacteria”) AND (“urinary bladder cancer”[MeSH] OR “bladder cancer” OR “bladder carcinoma” OR “urothelial carcinoma”).”

The reference for Shi et al. (reference 47) was erroneously written as:

“Shi Q, Geng J, Han L, Ji X, Li J, Mu Y, et al. Engineered Salmonella carrying siRNA-PD-1 shrinks orthotopically implanted bladder cancer in rats. J Drug Target. (2025), 1–9. doi: 10.1097/md.0000000000009292”

It should be:

“Shi Q, Geng J, Han L, Ji X, Li J, Mu Y, et al. Engineered Salmonella carrying siRNA-PD-1 shrinks orthotopically implanted bladder cancer in rats. J Drug Target. (2025), 1–9. doi: 10.1080/1061186X.2025.2512619”

The publisher apologizes for the error and the original version of this article has been updated.

